# Extensive phenotyping of vascular damage in non-infectious primary vasculitides with the use of non-invasive vascular biomarkers: prevalence, pathogenesis and response to treatment

**DOI:** 10.31138/mjr.29.3.173

**Published:** 2018-09-27

**Authors:** Ourania Agryropoulou, Athanase Protogerou, Antonis Argyris, Athanasios Tzioufas, Panayiotis Vlachoyiannopoulos

**Affiliations:** Department of Pathophysiology, “Laiko” Hospital, Medical School, National and Kapodistrian University of Athens, Athens, Greece

**Keywords:** Non-infectious primary vasculitides, vascular damage, arterial stiffness, atheromatosis, arterial remodeling, arterial hypertrophy, arteriosclerosis, cardiovascular risk factors, inflammation, anti-inflammatory treatment

## Abstract

Non-Infectious Primary systemic vasculitides (NIPSV) encompass a subset of autoimmune diseases, characterized mainly by intramural inflammation of the vascular wall. The increased mortality that some exhibit is partially attributed to vascular complications involving both micro- and macro- circulation. Beyond the disease specific pathways of vascular damage, emerging evidence suggest that the classical pathways of arterial damage, namely, atheromatosis, inappropriate arterial remodeling and arteriosclerosis are accelerated in several NIPSV; thus participating in the development of vascular complications in NIPSV patients. The aim of the current research protocol is to optimize the understanding of vascular pathology in NIPSV and to identify useful, easy to measure, non-invasive vascular tools for the diagnosis and follow-up of NIPSV patients. Moreover, the study aims to generate hypothesis regarding the molecular basis of the association of inflammation with classical vascular pathology.

## INTRODUCTION

Non-Infectious Primary Systemic Vasculitides (NIPSV) is a heterogeneous group of rare and potentially life-threatening diseases characterized by inflammation of the vascular wall.^1,2^ The size and localization of the involved vessels in association with the nature of the inflammatory process (focal or systemic, presence of necrosis, immune complex formation) account for the variability of the clinical manifestations between the various NIPSV.^2^ Prior to the introduction of corticosteroids, the natural history of untreated NIPSV was that of a rapidly progressive and usually fatal disease.^3,4^ Nowadays, the causes of death include cancer and infections due to chronic immune activation and/or to immunosuppressive therapy.^4,6^ Vascular complications (involving both micro- and macro-circulation) are major sources of morbidity and mortality in NIPSV. Microvascular complications are major causes of premature deaths especially in small vessel vasculitides: they affect predominantly the kidney (acute renal failure) and the lung (pulmonary hemorrhage).^5^ Macrovascular complications (e.g., coronary artery disease, stroke, aneurysm formation and rupture) are major causes of morbidity and mortality especially in medium and large vessel vasculitides.^3,4,5^ Lumen stenosis, occlusion or aneurysmal dilatation of blood vessels due to intramural inflammation and necrosis represent the major vascular pathology in NIPSV.^3^ Mural fibrin deposition in arterioles or venules, as well as angiocentric inflammatory cell infiltration are the hallmarks of biopsy-proven diagnosis.^3^ The early steps in the immunological process of vascular damage in NIPSV cannot be considered to be uniform, since various discrete mechanisms such as endothelial activation and dysfunction, autoantibodies to endothelial cell-surface antigens or neutrophil components and abnormal IgA tissue deposition are involved.^5^

Beyond the NIPSV specific pathways of vascular damage, numerous evidence suggests that the classical pathways of arterial damage, namely, atheromatosis (i.e., atheromatic plaque formation), inappropriate arterial remodeling (e.g., arterial hypertrophy / distention) and arteriosclerosis (i.e., arterial stiffening), are accelerated, thus participating in the development of micro- and macro-vascular complications in NIPSV.^4,7,8^ In brief, the main related mechanisms involve: (i) the primary intramural vessel wall inflammation; (ii) the secondary vessel wall inflammation due to systemic inflammation; (iii) NIPSV–related drug treatment induced deleterious effects on the vessel wall; and (iv) the effect of classical cardiovascular disease risk factors.^7^

However, numerous unanswered questions exist due to the limited number of NIPSV patients and methodological limitations (lack of prospective data, often misused interpretation of vascular biomarkers) that reduce the value of many published studies. Strong data on the acceleration of vascular damage (atheromatosis and increased arterial stiffening) are currently present in Takayasu Arteritis, Kawasaki Disease and Behcet’s Disease.^9^ The association of atheromatosis and arteriosclerosis with PN and small vessel vasculitides remains the less established, so far.^9^ The actual contribution of classical vascular pathology in the development of morbidity and mortality in NIPSV is not known. The effect of anti-inflammatory drugs on the function and structure of the large and small arteries has been scarcely evaluated in NIPSV. The potential clinical value of non-invasive vascular biomarkers such as carotid intimal-medial thickening and carotid-to-femoral pulse wave velocity prompts the need for large prospective cohorts in order to provide useful future guidance regarding the prognosis and treatment of PSV patients.^9^

## AIMS OF THE STUDY

*Primary aims:* (i) to identify the frequency of vascular damage, studied per pathology (atheromatosis, arteriosclerosis, remodeling) in the overall population, as well as, per disease, and compare it to the corresponding frequency of an age- and gender-matched control group; (ii) to identify vascular phenotypes, i.e., patterns of vascular damage (micro- and macro- circulation) per disease as well per vascular bed within each disease, as well as differences between the diseases; (iii) to quantify the relative contribution of each one of the above proposed mechanisms in the development of vascular damage in NIPSV; (iv) to identify vascular biomarkers (e.g., as the previously proposed “augmentation index”) that associate with disease activity in NIPSV; and (v) to study the effect of anti-inflammatory treatment from disease onset (or relapse) to complete remission and follow-up. To test the previously described hypothesis of biphasic effect of corticosteroids on the function and structure of the large and small arteries.

*Secondary aims:* (i) to identify the prevalence of classical cardiovascular diseases risk factors (hypertension, dyslipidemia, smoking, diabetes) in NIPSV, using state-of-the-art methodology (e.g., out-of-office blood pressure monitoring); (ii) Explorative & hypothesis generating analysis: DNA and miRNA will be extracted to stored. NIPSV considered as prototype of high grade vascular wall inflammation with extreme flares and periods of complete remission, will be used as a model to perform exploratory analysis leading to novel hypothesis regarding the molecular pathogenesis vascular disease linked to inflammation. Future perspectives (in a *separate 2nd phase study):* In a future follow-up study of the present cohort, we will evaluate the prognostic value of these biomarkers to predict morbidity and mortality and to be used as valid biomarkers to guide diagnosis and response to treatment.

## METHODS

Prospective observational study; anticipated study duration (based on 6 month pilot study is calculated around 3 years (2018–2021); sample size: 200 (25 per disease group at least) patients and 400 age- and gender-matched individuals without chronic inflammatory disease, NSIP or history of neoplastic disease who will serve as controls. Baseline and follow-up vascular evaluation of all the participants will be performed in pre-defined visits on the basis of disease activity, remission and relapse, as defined by international guidelines per disease (**Table 1**). Patients with any type of NIPSV - diagnosed on the basis of classical international criteria per disease - fulfilling the inclusion criteria will be recruited in one of the following 3 groups: Group A: consecutive newly diagnosed patient with NIPSV; Group B: NIPSV with active relapse; Group C: consecutive NIPSV patients in steady disease - remission status for at least 3 months with steady medication. Age- and gender-matched individuals without any chronic inflammatory disease and NIPSV will serve as control group. Extensive vascular studies with high resolution ultrasound, oscillometry and tonometry will be performed at the carotid bed, the femoral bed, the aorta, the upper arm and lower limbs and retina in order to evaluate atheromatosis, arteriosclerosis/elasticity, arterial remodeling and hypertrophy endothelial function, wave reflections and aortic hemodynamics, retinal geometry (**Table 2**) in 2 to 5 sequential visits within 6–9 months (visit 0, visit 1=1 week, visit 2= 1 month, visit 3= 3 months, visit 4 ≥ 6 months) in each patient starting from disease onset/relapse to complete remission. Anthropometric parameters, dietary habits, lipids and other blood samples, DNA, RNA, urine samples, tissues biopsies, PET/CT will be recorded in predefined visits.

**Table 1:** Study flow chart

Visit	Hospitalized	Duration	Anticipated disease status	NIPSV-Related treatment		Cardiovascular treatment	Vascular tests	PET/CT^*^	Blood tests^**^	Biopsies
				Cortico-steroids	Immuno-suppressive treatment					
0	yes	0	Active Disease (newly diagnosed or disease relapse)	Naive or steady for months	Naive or steady	Steady if possible	x	x	x	x If possible
1	yes	<7 days	Still active disease (newly diagnosed or relapse)	1–2 just after IV high dose completion therapy	1–2 days after 1^st^ dose	Steady if possible	x	+/-	x	+/-
2	no	1^st^ month	«Start» of remission	Just before dose tapering	Steady if possible	Steady if possible	x		x	
3	no	3^rd^ month	Remission	Tapering completion (start of minimal dose or no dose)	Steady if possible	Steady if possible	x		x	
4	no	6^th^ month	Remission	Steady	Steady if possible	Steady if possible	x		x	

**Legend**

Visit 0 for newly diagnosed patient (group A); visit 1 for disease relapse (group B); visit 3 for patient in steady disease remission status for at least 3 months with steady drugs (group C). ^*^ PET/CT will be performed at the Biomedical Research Foundation Academy of Athens (BRFAA) only for visits 0 or 1 and to verify complete disease remission (visit 4). ^**^ Renal function, metabolic profile, inflammation profile and disease specific test if need will be performed.

Serum from all visits will be stored anonymous at the biobank of cells and tissues of the Department of Pathophysiology of Medical School of National & Kapodistrian University of Athens.

**Table 2:** Assessment of macro- and micro-circulation.

Macrocirculation - *vascular bed, vascular-hemodynamic properties and non-invasive vascular biomarkers that will be assessed* Carotid to femoral pulse wave velocity (cfPWV) by pulse wave analysis (PWA (elastic arteries arteriosclerosis); Sphygomocr device Carotid to radial PWV by PWA (muscular arteries arteriosclerosis); high resolution ultrasound; GE Logic V5 Carotid elasticity (elastic arteries arteriosclerosis) (right and left); high resolution ultrasound; GE Logic V5 Carotid artery wall to lumen ratio (arterial remodelling) (right and left); high resolution ultrasound; GE Logic V5 Carotid artery intimal-medial thickness (IMT) (right and left)(arterial hypertrophy / atheromatosis); high resolution ultrasound; GE Logic V5 Carotid (common, bulb, internal; right and left) bed plaques (atheromatosis); high resolution ultrasound; GE Logic V5 Femoral bed-plaques (atheromatosis); high resolution ultrasound; GE Logic V5 Ankle-brachial index (atheromatosis and arteriosclerosis); oscillometric device; Micrlife office BP - ABI. Brachial artery flow-mediated dilatation (endothealial function); high resolution ultrasound; GE Logic V5 Abdominal aorta and subclavian artery diameters; high resolution ultrasound; GE Logic V5 Aortic blood pressure; subendocardial viability index by PWA; Sphygomocr device Twenty-four hours ambulatory aortic stiffness, aortic blood pressure, brachial blood pressure, cardiac output and total prepheral resistance monitoring; Mobilograph IEM device Retinal microcirculation by digital camera photography (and dedicated software analysis (Imedos) Pressure wave reflections (augmentation index) by PWA; Sphygomocr device Pressure wave reflections by twenty-four hourw ambulatory aortic hemodynamic monitoring; Mobilograph IEM device

Patients involved in the study are informed in detail and give written consent. All data are collected under code in anonymous electronic files, in which only researchers have access, at the Cardiovascular Prevention & Research Unit of the Department of Pathophysiology of Medical School of National & Kapodistrian University of Athens. Under the above particular circumstances, biological materials; blood serum, DNA, RNA and biopsy tissues are collected and retained anonymous at the biobank of cells and tissues of the Department of Pathophysiology of Medical School of National & Kapodistrian University of Athens. Ethical approval has been obtained by the Bioethics and Ethics Committee of National and Kapodistrian University of Athens.

### Statistical analysis

Statistical analysis will be performed by SPSS v. 23.0 using appropriate test per hypothesis and populations. The data will be analyzed and presented in the overall population as well per disease and disease status, after normal distribution control. Comparison of the outcome variables (vascular indices) between and within groups will be performed before and after adjusting for potential confounders using independent multiple t-tests, ANOVA, paired t-test, linear and logistic regression analysis, and chi-square tests as appropriate. Receiver operator curve analysis will be performed to identify ability of the outcome variables to detect disease activity. Sensitivity and mediation analysis will be performed.

## ANTICIPATED BENEFITS:

- To optimize the understanding of vascular pathology in NIPSV.
- To identify potential useful easy to measure non-invasive vascular tools for diagnosis and follow-up of NIPSV patients.
- To generate hypothesis regarding the molecular basis of association of inflammation with classical vascular pathology (atheromatosis, arteriolosclerosis, arterial remodeling).

## CONFLICT OF INTEREST

The authors declare no conflict of interest.
